# Size Limit and Energy Analysis of Nanoparticles during Wrapping Process by Membrane

**DOI:** 10.3390/nano8110899

**Published:** 2018-11-02

**Authors:** Xinpei Meng, Xinlei Li

**Affiliations:** 1 Mechatronics Technology R&D and Service Center, Dongguan Polytechnic, Dongguan 523808, China; mengxp@dgpt.edu.cn; 2 MOE Key Laboratory of Laser Life Science & Institute of Laser Life Science, College of Biophotonics, South China Normal University, Guangzhou 510631, China

**Keywords:** wrapping process, endocytosis, nanoparticle, thermodynamics, cell membrane

## Abstract

The wrapping of nanoparticles (NPs) by a membrane is a phenomenon of widespread and generic interest in biology, as well as in a variety of technological applications, such as drug delivery, clinical diagnostics, and biomedical imaging. However, the mechanisms of the interaction between the membrane and NPs are not well understood yet. In this paper, we have presented an analytic thermodynamic model to investigate the wrapping process of NPs by a cell membrane. It is found that the bending energy of the deformed membrane increases nonlinearly with increasing wrapping degree, which leads to a free energy barrier for the wrapping. On the basis of analysis results, the wrapping of NPs can be divided into three types, i.e., impossible wrapping, barrier wrapping, and free wrapping. Furthermore, a phase diagram for the wrapping of NPs has been constructed, which clarifies the interrelated effects of the size and the ligand density of NPs. We hope that this work can provide some help in understanding the physical mechanism of the wrapping of NPs.

## 1. Introduction 

Nanometer-sized particles have been a focus of intensive research in the biomedical field for applications such as drug delivery, clinical diagnostics, and biomedical imaging [1,2,3,4,5,6]. In order to raise diagnostic sensitivity and therapeutic efficiency, the ability to control and manipulate the interaction between cell membrane and nanoparticles (NPs) is required. Generally, NPs can enter a cell through wrapping by a cell membrane, i.e., cell endocytosis [7,8,9,10,11]. Experimental studies on site-targeted drug delivery into cells have revealed that the wrapping process is critically dependent on particle size [11,12,13,14,15]. Meanwhile, various models have been established to pursue the mechanism involved in the receptor-mediated wrapping process of NPs based on thermodynamics and dynamics [7,8,9,10,15,16,17,18,19,20]. Theoretical research showed that wrapping proceeds only if the chemical binding energy density of NPs with a membrane exceeds the bending energy density of the deformed membrane [9,10,15]. Therefore, there exists a minimal size of NPs for wrapping to occur. Below this minimal size, NPs cannot be internalized through receptor-mediated endocytosis. Above the minimal size, wrapping is energetically favorable. In spite of much progress in the theory and research to date, the particular mechanisms of the wrapping process of NPs by membrane remain unclear. For instance, how do the NP and membrane behave during the wrapping process? Is there a connection between wrapping degree and the level of difficulty of cellular internalization? 

Based on the above issues, in this contribution we propose an analytic model to study the wrapping process of NPs by cell membrane through cell endocytosis, including the geometry of a deformed membrane and the free energy change with wrapping degree. Our theoretical results reveal that the bending energy of the membrane leads to a barrier wrapping of NP. Furthermore, we construct a phase diagram for the wrapping of NPs in the space of radius and ligand density. The phase diagram may provide firsthand guidance for the design of NPs for diagnostic agents and drug delivery applications. 

## 2. Theoretical Model 

**Figure 1:**
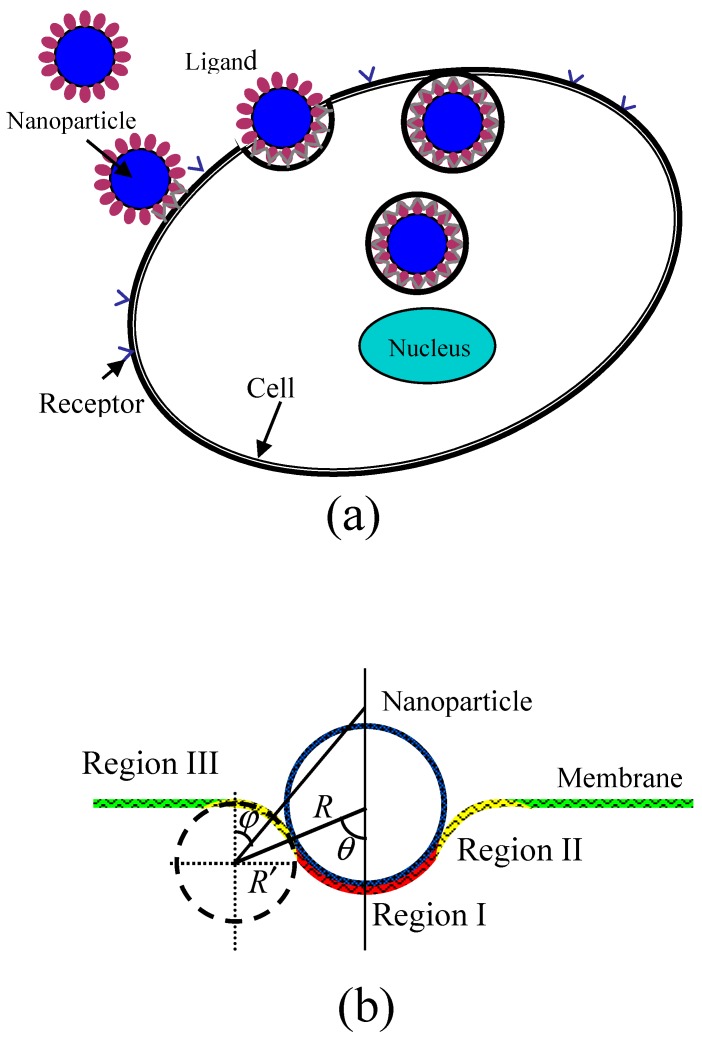
(**a**) Schematic illustration of nanoparticles (NPs) internalized by cell membrane to different degrees: away from the membrane, the partially wrapped state and fully wrapped state. (**b**) Schematic geometry of a particle wrapped by an initially flat membrane.

During the wrapping process of a rigid spherical particle by a membrane, as shown in Figure 1, the membrane is divided into three regions. The first is the bound region (Region I) of the membrane in contact with the particle, where receptors on the surface of membrane are packed on the surfaces of NPs through binding with ligands, causing a strong change of curvature of the membrane. The second is the deformed region nearly in contact with the particle (Region II) which causes an increase of curvature. The last is the undeformed region (Region III) of the membrane which still maintains its original curvature. Figure 1b shows a schematic of the partially wrapped state of a spherical particle by a membrane. When the receptor-mediated endocytosis of NPs occurs, the driving force is the energy releases as chemical bonds form when receptors change from free to bound states. Further, the process can also lead to the loss of configurational entropy of the receptors. The deformation of the cell membrane results in the increase of energy as the bending energy which prevents particle from wrapping. Considering the three contributions to the wrapping by the membrane and using $k_{B}T$ (where $k_{B}$ is the Boltzmann constant and *T* is absolute temperature) as unit of energy, we can write the free energy change during the wrapping process as:

(1) $$\begin{array}{l} \Delta E/k_{B}T=S_{b}\left\{\left[\xi_{b}\ln\xi_{b}+\left(1-\xi_{b}\right)\ln\left(1-\xi_{b}\right)\right]-\left[\xi_{f}\ln\xi_{f}+\left(1-\xi_{f}\right)\ln\left(1-\xi_{f}\right)\right]\right\}-\mu S_{b}\xi_{b}\\ +\int_{S_{b}}\left[\frac{\kappa }{2}{\left(c_{b}{}_{1}+c_{b}{}_{2}-c_{0}\right)}^{2}\right]d^{2}A+\int_{S_{f}}\left[\frac{\kappa }{2}{\left(c_{f}{}_{1}+c_{f}{}_{2}-c_{0}\right)}^{2}\right]d^{2}A \end{array}$$

where $S_{b}$ represents the area of the bound membrane region, $\xi_{b}$ and $\xi_{f}$ are the densities of bound receptors in Region I and free receptors in Regions II and III, μ is the released chemical energy caused by a ligand-receptor chemical binding, κ is the bending modulus of the membrane, $c_{b}{}_{1}$ and $c_{b2}$ are two principal curvatures of the bound membrane surface, $c_{0}$ is the spontaneous curvature, and $c_{f}{}_{1}$ and $c_{f2}$ are two principal curvatures of the deformed membrane surface in Region II. The first term in Equation (1) represents the contributions of the translational entropies of the bound and free receptors [21,22], the second term represents the release of chemical binding energy, and the last two terms represent the deformed bending energy of the membrane in Region I and II in which $c_{b}{}_{1}$, $c_{b2}$, and $c_{f2}$ are positive (convex viewed from the inside of the cell), and $c_{f2}$ is negative, respectively. 

Wrapping of a particle occurs spontaneously only when the free energy continuously decreases as contact bound area ($S_{b}$) increases. Therefore, we can analyze the values of free energy change caused by particle wrapping using Equation (1). Before calculating the free energy change, we should first determine the geometry of the deformed membrane, i.e., the bending energy of the deformed membrane. In our model, we assume that the vertical cross section of membrane in Regions I and II has radii of curvature of R and $R^{\prime }$, respectively, as shown in Figure 1b. Further, the initial membrane is considered to be planar with a spontaneous curvature of zero because the size of NPs is much smaller than that of the membrane. More specifically, the size of a typical cell is about 1–10 μm, which is significantly larger than that of NPs (which are smaller than 100 nm), which means that the curvature of NPs is at least ten times larger than the spontaneous curvature of the initial membrane. In this case, the spontaneous curvature of the initial membrane can be ignored. On the other hand, we must consider the contribution of the spontaneous curvature of the initial membrane if it has the same order of magnitude as that of the NPs. In our model, therefore, both of the two principal curvatures of the bound membrane surface (Region I) are 1/R. Therefore, the bending energy of the membrane in Region I by the Canham-Helfrich Hamiltonian equation is [23,24]:

(2) $$\int_{S_{b}}\left[\frac{\kappa }{2}{\left(c_{b}{}_{1}+c_{b}{}_{2}-c_{0}\right)}^{2}\right]d^{2}A=\int_{S_{b}}\left[\frac{\kappa }{2}{\left(\frac{2}{R}-c_{0}\right)}^{2}\right]d^{2}A=4\pi \kappa \left(1-\cos\theta \right)\;$$

where θ is the contact angle of the particle with deformed membrane, and $S_{b}=2\pi R^{2}\left(1-\cos\theta \right)$, as shown in Figure 1. We define angle θ as the degree of wrapping. When the particle is completely unwrapped, the angle θ is zero. 

The membrane surface in Region II has two different principal curvatures. The first is the curvature of the vertical cross section of membrane, $-1/R^{\prime }$, where the negative sign represents the concave surface. The other is the curvature of $c_{f2}$, which is determined by the location on the membrane surface and can be calculated by $c_{f}{}_{2}=\sin\phi /\left[\left(R+R^{\prime }\right)\sin\theta -R^{\prime }\sin\phi \right]$, where ϕ is the angle between a vertical line on the undeformed membrane surface and the line joining the center of the vertical cross section of membrane and the local location on the deformed membrane surface, as shown in Figure 1. So, the bending energy of the deformed membrane in Region II can be expressed as:

(3) $$\begin{array}{l} \int_{S_{f}}\left[\frac{\kappa }{2}{\left(c_{f}{}_{1}+c_{f}{}_{2}-c_{0}\right)}^{2}\right]d^{2}A\\ =\int_{S_{f}}\left[\frac{\kappa }{2}{\left(-\frac{1}{R^{\prime }}+\frac{\sin\phi }{\left(R+R^{\prime }\right)\sin\theta -R^{\prime }\sin\phi }\right)}^{2}\right]d^{2}A\\ =\int_{0}^{\theta }\left\{\frac{\kappa }{2}{\left[-\frac{1}{R^{\prime }}+\frac{\sin\phi }{\left(R+R^{\prime }\right)\sin\theta -R^{\prime }\sin\phi }\right]}^{2}2\pi \left[\left(R+R^{\prime }\right)\sin\theta -R^{\prime }\sin\phi \right]R^{\prime }\right\}d\phi \\ =\pi \kappa \int_{0}^{\theta }\left[\frac{{\left(\frac{R}{R^{\prime }}+1\right)}^{2}\sin^{2}\theta }{\left(\frac{R}{R^{\prime }}+1\right)\sin\theta -\sin\phi }-4\sin\phi \right]d\phi \end{array}$$

When $\left(\frac{R}{R^{\prime }}+1\right)\sin\theta <1$ and if $M=\left(\frac{R}{R^{\prime }}+1\right)\sin\theta$, we obtain:

(4) $$\begin{array}{l} \int_{S_{f}}\left[\frac{\kappa }{2}{\left(c_{f}{}_{1}+c_{f}{}_{2}-c_{0}\right)}^{2}\right]d^{2}A\\ =\pi \kappa \left[\frac{M^{2}}{\sqrt{1-M^{2}}}\left(\ln\left|\frac{M\tan\frac{\theta }{2}-1-\sqrt{1-M^{2}}}{M\tan\frac{\theta }{2}-1+\sqrt{1-M^{2}}}\right|-\ln\left|\frac{1+\sqrt{1-M^{2}}}{1-\sqrt{1-M^{2}}}\right|\right)-4\left(1-\cos\theta \right)\right] \end{array}$$

When $\left(\frac{R}{R^{\prime }}+1\right)\sin\theta >1$, we have:

(5) $$\int_{S_{f}}\left[\frac{\kappa }{2}{\left(c_{f}{}_{1}+c_{f}{}_{2}-c_{0}\right)}^{2}\right]d^{2}A=\pi \kappa \left[\frac{2M^{2}}{\sqrt{M^{2}-1}}\left(\arctan\frac{M\tan\frac{\theta }{2}-1}{\sqrt{M^{2}-1}}-\arctan\frac{-1}{\sqrt{M^{2}-1}}\right)-4\left(1-\cos\theta \right)\right]\;$$

## 3. Results and Discussion

**Figure 2:**
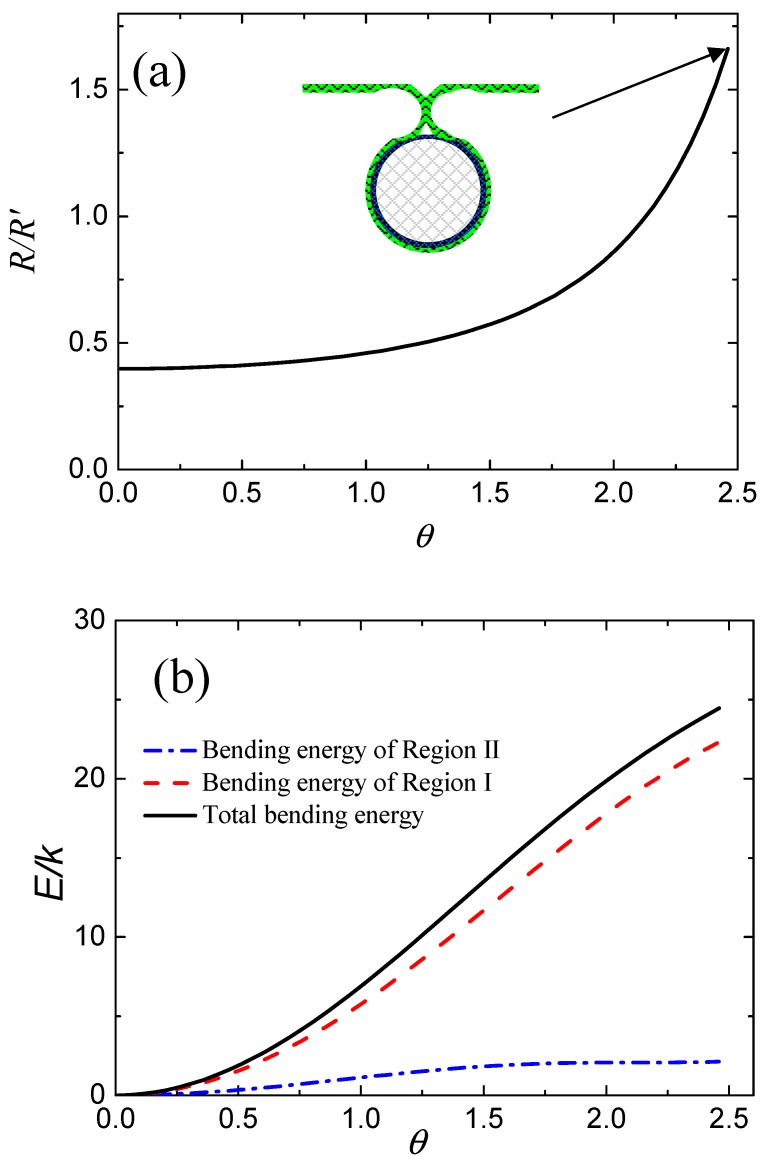
(**a**) The calculated values of $R/R^{\prime }$ as a function of the wrapping degree in the equilibrium state based on Equations (4) and (5). (**b**) The calculated values of total bending energy and partial bending energies of the deformed membrane in Region I and Region II.

According to Equations (4) and (5), we find that the bending energy of membrane in Region II is a function of $R/R^{\prime }$ and θ. Therefore, we can obtain the equilibrium shape of the deformed membrane by minimizing the bending energy for different wrapping degrees. Figure 2a shows the variations in $R/R^{\prime }$ with respect to wrapping degree in the equilibrium state based on Equations (4) and (5). We note that the value of $R/R^{\prime }$ increases with increasing wrapping degree, θ, and reaches a maximum until the deformed membrane touches. It should be noted that the value of $R/R^{\prime }$ in Figure 2a is vacant because both R and $R^{\prime }$ are infinite when θ=0. More specifically, both R and $R^{\prime }$ approach infinity before contact of the membrane, and thus the value of $R/R^{\prime }$ is meaningless. Figure 2b shows the calculated values of total bending energy of the membrane and partial bending energies of the deformed membrane in Region I and Region II. We find that the values of bending energy of the membrane in Region I and Region II always increase with increasing θ, as shown by the red dashed line and the blue dotted line in Figure 2b. Therefore, the total bending energy of the deformed membrane—i.e., the sum of these two terms (black line in Figure 2b)—has the same change tendency, i.e., to increase with increasing θ. 

**Figure 3:**
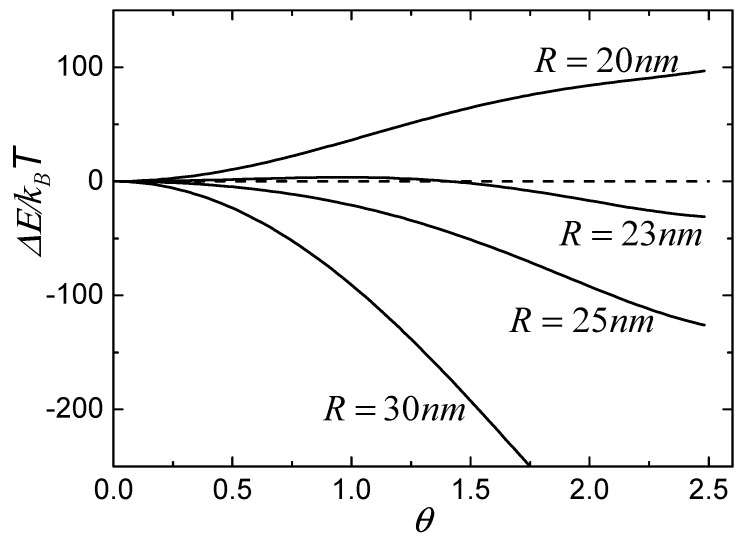
Free energy change, ΔE, as a membrane wraps around a spherical particle with wrapping degree θ for different radii of NPs.

After we know the bending energy of the deformed membrane, we can calculate the total free energy change during the wrapping process based on Equation (1). Figure 3 shows the calculated values of free energy change as a function of the wrapping degree, θ, for different NPs with radii of 20 nm, 23 nm, 25 nm, and 30 nm. In the calculations, we defined $\mu =20k_{B}T$ [7] and $\kappa =20k_{B}T$ [24]. The density of free receptors is considered to have the same value as that of initial receptors, that is, $\xi_{0}=0.05$ [25,26], and the density of bound receptors in Region I is equal to the density of ligands on the surface of particles, so that $\xi_{l}=1$. From Figure 3, we can classify the wrapping conditions of particles into three types according to the tendency of free energy change. The first type is such that the free energy change is always less than zero and decreases with increasing θ, as shown by the lines of particles of 25 nm and 30 nm, which suggests that particles with large radii can be continuously wrapped by the membrane. The second type is such that the free energy change is always larger than zero when the radius of particle is smaller than a certain value, which suggests that the small particle cannot be wrapped, as shown by the line of the particle of 20 nm. The last type is such that the free energy change is first larger than zero and then decreases to less than zero with increasing θ, as shown by the line of the particle of 23 nm, which indicates that wrapping of the particle is unfavorable initially, but then becomes energetically favorable when the free energy change is less than zero with increasing θ. In other words, the membrane can wrap the particle only if the process overcomes an energy barrier. 

Based on the above analysis, we can obtain two critical sizes of particles for wrapping by the membrane. The first critical size corresponds to the condition that the free energy change is always less than zero, and the other critical size corresponds to the condition that the free energy change becomes less than zero when the wrapping degree θ reaches its maximum. When the free energy change during the wrapping process is equal to zero, based on Equation (1), we can obtain:

(6) $$r_{c}=\sqrt{\frac{\int_{S_{b}}\left[\frac{\kappa }{2}{\left(c_{b}{}_{1}+c_{b}{}_{2}-c_{0}\right)}^{2}\right]d^{2}A+\int_{S_{f}}\left[\frac{\kappa }{2}{\left(c_{f}{}_{1}+c_{f}{}_{2}-c_{0}\right)}^{2}\right]d^{2}A}{2\pi \left(1-\cos\theta \right)\left\{\mu \xi_{b}+\left[\xi_{f}\ln\xi_{f}+\left(1-\xi_{f}\right)\ln\left(1-\xi_{f}\right)\right]-\left[\xi_{b}\ln\xi_{b}+\left(1-\xi_{b}\right)\ln\left(1-\xi_{b}\right)\right]\right\}}}\;$$

According to Equations (2)–(5), we can calculate the maximum and minimum values of $\left\{\int_{S_{b}}\left[\frac{\kappa }{2}{\left(c_{b}{}_{1}+c_{b}{}_{2}-c_{0}\right)}^{2}\right]d^{2}A+\int_{S_{f}}\left[\frac{\kappa }{2}{\left(c_{f}{}_{1}+c_{f}{}_{2}-c_{0}\right)}^{2}\right]d^{2}A\right\}/\left[2\pi \left(1-\cos\theta \right)\right]$, respectively, as $2.46A_{0}\kappa$ and $2.19A_{0}\kappa$ when wrapping degree θ goes to 0 and its maximum. Therefore, we can get the first critical radius of NPs as:

(7) $$r_{c1}=\sqrt{\frac{2.46A_{0}\kappa }{\mu \xi_{b}+\left[\xi_{f}\ln\xi_{f}+\left(1-\xi_{f}\right)\ln\left(1-\xi_{f}\right)\right]-\left[\xi_{b}\ln\xi_{b}+\left(1-\xi_{b}\right)\ln\left(1-\xi_{b}\right)\right]}}\;$$

The second critical radius of particles is:

(8) $$r_{c2}=\sqrt{\frac{2.19A_{0}\kappa }{\mu \xi_{b}+\left[\xi_{f}\ln\xi_{f}+\left(1-\xi_{f}\right)\ln\left(1-\xi_{f}\right)\right]-\left[\xi_{b}\ln\xi_{b}+\left(1-\xi_{b}\right)\ln\left(1-\xi_{b}\right)\right]}}\;$$

When the radius of NPs is larger than $r_{c1}$, the free energy change is always less than zero and decreases with increasing θ, which shows that these large NPs can be wrapped favorably by the membrane. When the radius of particles is smaller than $r_{c1}$ but larger than $r_{c2}$, the free energy change is first larger than zero and then decreases to less than zero with increasing θ, which suggests that the wrapping of particle is unfavorable initially, but then becomes favorable with increasing wrapping degree θ. However, when the radius of particles is smaller than $r_{c2}$, the free energy change is always larger than zero, and these small NPs cannot be wrapped by the membrane. 

**Figure 4:**
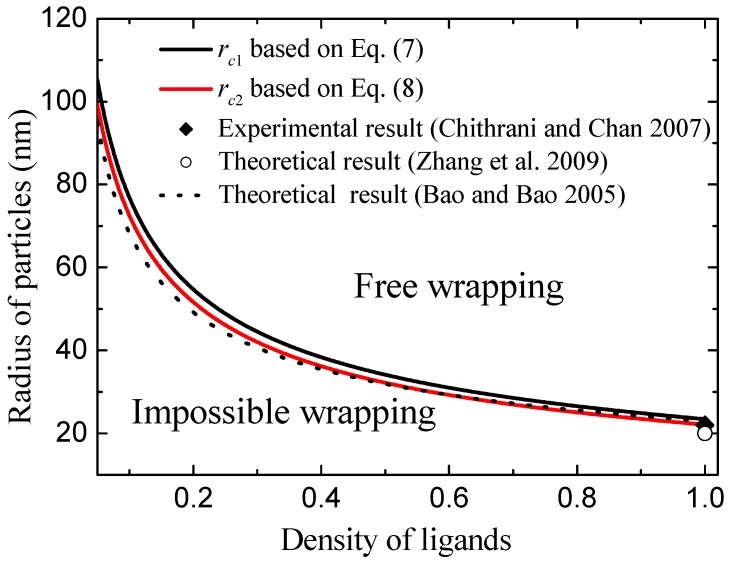
The calculated critical radius of $r_{c1}$ and $r_{c2}$ as a function of ligand density using Equations (7) and (8), and comparisons with experimental and other theoretical results.

Figure 4 shows the calculated critical radius of $r_{c1}$ and $r_{c2}$ as the function of ligand density using Equations (7) and (8). Clearly, we can see that the low density of ligands leads to the values of $r_{c1}$ and $r_{c2}$ increasing. The physical mechanisms are such that the low density of ligands induces a weak chemical binding of ligands with receptors. Weak chemical binding needs a greater binding area in order to oppose the bending energy of deformed membrane. Because bending energy is independent of the radius of the NP, increasing particle size can result in the increase of binding area. Therefore, the low density of ligands leads to high values of $r_{c1}$ and $r_{c2}$. Based on Figure 4, we divide the wrapping condition (radius of NPs, *R*, and ligand density, $\xi_{l}$) into three zones according to the values of $r_{c1}$ and $r_{c2}$. When the radius of the nanoparticle is less than $r_{c2}$, the particle cannot be wrapped by the membrane. We name this condition impossible wrapping. When the radius of the nanoparticle is larger than $r_{c2}$ but less than $r_{c1}$, the membrane can wrap the particle when the process overcomes an energy barrier. We name this condition barrier wrapping. When the radius of the nanoparticle is larger than $r_{c1}$, the free energy change is always less than zero and the large particle can be wrapped favorably by the membrane. This condition is named free wrapping. More importantly, these theoretical results are consistent with experiments [11] and other theoretical results [7,10,18], as shown in Figure 4. For example, Chithrani et al. observed that endocytosis of gold NPs of 7 nm radius into animal cells can only occur when at least six of the NPs cluster together. This cluster radius is of the order of about 20 nm [11]. Theoretically, Zhang et al., calculated the minimum radius of NPs is about 22 nm for endocytosis [7]. These minimum radii are consistent with our results of $r_{c1}=23.5\mathrm{nm}$ and $r_{c2}=22.1\mathrm{nm}$ for $\xi_{l}=1$. In addition, the values of minimum radius of NPs for endocytosis calculated by Bao et al. [10] agree well with our model prediction, as shown by the dashed line in Figure 4. 

Based on the established model, we can know that the size limit of NPs during the wrapping process is strongly affected by the densities of receptors, the released chemical energy caused by ligand-receptor chemical binding (μ), and the rigidity of cell membranes (i.e., bending modulus). From Equations (7) and (8), we find that low densities of receptors and small μ result in a large critical size of NPs. Similar results and discussions have also been reported in our previous work [22]. In extraordinary instances of a receptor-free membrane, the loss of configurational entropy of the receptors in Equation (1) should be ignored, and the wrapping process is driven by the adhesion energy between the membrane and NP. In this case, the critical size of NPs becomes much larger than the case in our model because the adhesion energy is much smaller than the released chemical energy (μ). On the other hand, we consider that ligand-receptor chemical binding is instantaneous and much faster than the diffusion of receptors. Therefore, receptor density in the Regions I-II-III is considered to be a constant during the wrapping process and the relative dynamical process is neglected in our model [7,8,22]. Besides the effects of the densities of receptors and the released chemical energy, the rigidity of cell membranes—i.e., bending modulus (κ)—also determines the size limit of NPs. The value of bending modulus used in our calculations is that of typical cancer cell [24]. For different cells, as expressed in Equations (7) and (8), a large bending modulus of the membranes results in a large critical radius of NPs because large bending modulus means strong resistance and difficulty for wrapping. It should be noted that most NPs used in nano medicine are usually in the 180–250 nm range, which is significantly larger than the critical radius of NPs calculated in our model. This means that NPs with large sizes can be fully wrapped by cell membranes. However, NPs cannot been wrapped if their size is less than the critical radius according to our modeling results. 

Furthermore, we only consider the wrapping process of a single spherical NP in our model. The effects of membrane curvature, particle shape and orientation, and interactions between NPs on the wrapping process are not considered. Fortunately, the effects of these factors have been discussed in the existing literature [27,28,29,30,31]. Remarkably, Tang et al. recently formulated a model to theoretically investigate the effects of the particle shape and orientation, and interactions between NPs, on wrapping process. Their results revealed the roles of the shape, rotation, and initial orientation of the NP, and interactions between NPs, on receptor-mediated endocytosis, and provide good guidelines for the design of NP-based drug delivery systems [30,31]. 

## 4. Conclusions 

In conclusion, we present an analytical model to study the wrapping process of NPs by cell membrane. Through investigating the geometry of a deformed membrane, we find that the bending energy of the deformed membrane increases with increasing wrapping degree, which leads to an energy barrier for wrapping. Therefore, according to the size of NPs and ligand density, the wrapping of NPs can be divided into three types, i.e., impossible wrapping, barrier wrapping, and free wrapping. A phase diagram for the wrapping of NPs in the space of radius and ligand density was constructed. Agreement between theoretical results and experimental observations implies that the proposed model could be applicable to understanding the basic physical mechanism of the wrapping of NPs.
